# Phylogeographic Insights into *Pipistrellus* Species from Türkiye: Diversity, Divergence, and Regional Lineage Structure

**DOI:** 10.3390/biology14111549

**Published:** 2025-11-04

**Authors:** Emin Seyfi, Şafak Bulut, Gül Olgun Karacan

**Affiliations:** 1 Institute of Science, Hitit University, 19030 Çorum, Türkiye; 2 Department of Biology, Faculty of Science, Gazi University, 06500 Ankara, Türkiye; safak82@gmail.com; 3 Vocational School of Health Services, Aksaray University, 68100 Aksaray, Türkiye; golgun@aksaray.edu.tr

**Keywords:** mitochondrial Cyt*b*, phylogeography, *Pipistrellus*, secondary contact zone, Türkiye

## Abstract

**Simple Summary:**

Bats are important mammals that contribute to natural balance by consuming insects and serving as indicators of healthy ecosystems. In Türkiye, four closely related species of the genus *Pipistrellus* are found, yet they are extremely difficult to identify based on external appearance alone. This study investigated their genetic diversity and evolutionary history using mitochondrial Cyt*b* sequences collected from a wide range of regions. The results showed that external features often lead to misidentification, especially between the common pipistrelle and the soprano pipistrelle, highlighting the importance of molecular data for reliable classification. We also discovered that Türkiye plays a dual role in bat evolution, serving both as a refugium for unique lineages and as a contact zone where different lineages meet. Distinct lineages of the common pipistrelle and Kuhl’s pipistrelle were found to overlap within Anatolia, and a previously unrecognized lineage of Nathusius’ pipistrelle was detected in southwestern Türkiye. In *Pipistrellus pipistrellus*, the Anatolian Diagonal separates eastern and western lineages while still allowing their overlap, acting as both barrier and corridor. These findings emphasize the value of Türkiye as both a refugium and a crossroads for bat diversity, providing knowledge essential for conservation and ecosystem protection.

**Abstract:**

This study investigates the phylogenetic relationships, genetic diversity, and biogeographic structure of *Pipistrellus* species in Türkiye using mitochondrial cytochrome *b* (Cyt*b*) sequences from 156 specimens collected across 26 localities. Our primary aim was to clarify taxonomic boundaries of morphologically cryptic species and elucidate the evolutionary role of Anatolia in the Western Palearctic. Analyses strongly confirmed that molecular data are mandatory for defining taxonomic boundaries. Crucially, all individuals morphologically identified as *P. pygmaeus* were genetically determined to be *P. pipistrellus*, highlighting the inadequacy of external traits for cryptic species. We resolved deep intraspecific divergence across the genus. In *P. pipistrellus*, two major lineages (Eastern and Western) were identified, partially separated by the Anatolian Diagonal. Their co-occurrence in multiple localities confirms Anatolia’s function as a secondary contact zone. Similarly, *P. kuhlii* populations represent a transition zone where two distinct lineages, one of Asiatic origin (*P. k. lepidus*) and one Mediterranean-Levantine (*P. k. kuhlii*), meet. Furthermore, while *P. nathusii* is largely associated with migratory European lineages; a genetically distinct, potentially resident lineage was revealed in southwestern Anatolia. Divergence time estimations indicate that this diversification was shaped by major climatic events from the Miocene to the Pleistocene. This study demonstrates that Anatolia is more than just a geographic bridge; it is a dynamic center of evolution, functioning critically as both a glacial refugium and a secondary contact zone for Palearctic bat fauna.

## 1. Introduction

The Mediterranean Basin is recognized as one of the world’s biodiversity hotspots, acting as a critical refugium for many taxa during the Quaternary glacial cycles [1]. Its complex topography and climatic heterogeneity fostered the survival, divergence, and secondary contact of numerous species lineages. Straddling the Asian and European continents, Türkiye acts as a natural bridge and biogeographic corridor [1,2]. This makes it an ideal landscape for studying evolutionary processes, including range shifts, species radiation, and gene flow. This strategic location and diverse ecological zones have crucially shaped the genetic and phenotypic diversity of its mammalian fauna. Bats (Chiroptera), which comprise nearly one-quarter of all mammal species worldwide, are particularly affected [3,4,5].

Within Chiroptera, the genus *Pipistrellus* (family Vespertilionidae) represents a taxonomically and ecologically diverse group, distributed across the Palearctic region and known for its cryptic morphology, high dispersal capabilities, and wide habitat tolerance [6,7]. The genus includes approximately 35 species globally. Four species—*P. pipistrellus* (Schreber, 1774), *P. kuhlii* (Kuhl, 1817), *P. nathusii* (Keyserling & Blasius, 1839), and *P. pygmaeus* (Leach, 1825)—are confirmed to occur in Türkiye [8]. These species are often sympatric, yet their identification remains problematic due to subtle morphological differences and the presence of cryptic species complexes [9,10,11,12].

Regional studies on Turkish *Pipistrellus* populations have focused on morphological [13,14], karyological [15], and ecological aspects [16,17]. However, despite the species’ broad distribution and ecological importance, molecular studies on their phylogenetics and phylogeography remain limited in geographic scope and depth. This gap is critical for conservation. Bats serve as keystone species yet are highly sensitive to anthropogenic pressures such as habitat fragmentation, deforestation, agricultural intensification, and wind energy infrastructure expansion [18,19,20,21].

Molecular phylogenetic approaches, especially those based on mitochondrial markers such as cytochrome *b* (Cyt*b*), have become fundamental tools for studying species boundaries, uncovering cryptic diversity, and reconstructing historical biogeographic scenarios [22,23,24,25,26,27]. Although our study focuses on mitochondrial data, such markers provide valuable first-line insights into genetic structure and biogeographic history, particularly when broader genomic data are not yet available. Moreover, the application of *Cytb* in Chiroptera phylogeography has enabled the identification of divergent lineages, postglacial recolonization routes, and historical refugia. For example, Hulva et al. showed that *P. pipistrellus* populations in Türkiye, especially those in Antalya and Cappadocia, have high haplotypic diversity [28]. This suggests these areas may be evolutionary hotspots. Similarly, Çoraman et al. analyzed *ND1* and *Cytb* across four Turkish *Pipistrellus* species [2]. They revealed distinct phylogeographic structures and potential secondary contact zones, particularly in Anatolia and the Black Sea region.

Despite these advances, the taxonomic and phylogeographic statuses of several Turkish *Pipistrellus* species and subspecies remain unresolved. For instance, Albayrak proposed a three-subspecies structure for *P. pipistrellus* based on morphometrics (*P. p. pipistrellus, P. p. aladdin*, and *P. p. mediterraneus*) [13]. However, this scheme lacks molecular validation. Furthermore, the altitudinal and latitudinal variation in *P. kuhlii* populations suggests subspecific divergence. Some researchers classify Turkish populations as *P. k. ikhwanius* or *P. k. lepidus*, based on morphological similarity to Arabian or Middle Eastern populations [17,29]. *P. nathusii*, known for its long-distance seasonal migration [30,31], has been recorded in Thrace and the eastern Black Sea region of Anatolia. This suggests Türkiye may serve as both a migratory corridor and a wintering ground [32]. Additionally, the taxonomic resolution of *P. pygmaeus*, a cryptic species historically conflated with *P. pipistrellus*, remains challenging in Türkiye. Molecular analyses confirm its presence [11,12]. Yet, recent studies, including this one, show persistent misidentification when relying solely on morphological traits [9,33].

In this study, we evaluate the genetic diversity, phylogenetic relationships, and biogeographic patterns of all currently documented *Pipistrellus* species in Türkiye using partial sequences of the mitochondrial cytochrome *b* (Cyt*b*) gene. We use an expanded sampling strategy across all major biogeographic regions of Türkiye and integrate our data with previously published European and Asian sequences. This study seeks to: (i) clarify the taxonomic boundaries among *Pipistrellus* species and subspecies in Türkiye, (ii) identify potential historical refugia and zones of secondary contact, (iii) elucidate broader phylogeographic structures of Palearctic-distributed taxa, and (iv) estimate the evolutionary divergence times among major lineages within the genus, providing a temporal framework for speciation and diversification.

## 2. Materials and Methods

### 2.1. Tissue Collection

**Figure 1:**
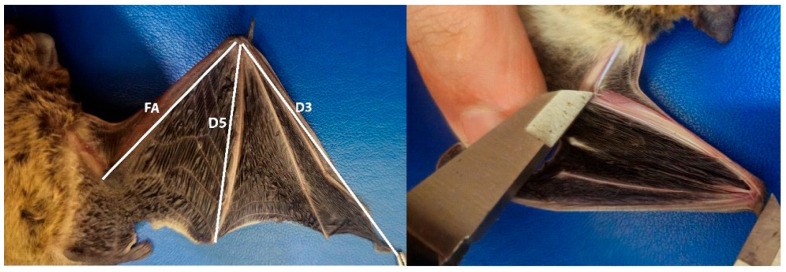
External morphometric measurements were taken from both carcass and live specimens of *Pipistrellus* species, including forearm length (FA), third digit length (D3), and fifth digit length (D5).

**Figure 2:**
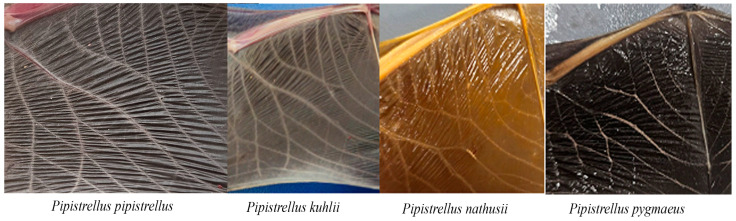
Illustrative diagrams of wing membrane patterns in different *Pipistrellus* species, based on diagnostic morphological traits described by Dietz and von Helversen [34].

Fieldwork was conducted across Türkiye to sample the distributional range of *Pipistrellus* species. Sampling targeted diverse habitats, including caves, tree hollows, and abandoned buildings. Roosting bats were commonly observed in rural settings, often found in roof spaces, window ledges, and electrical panels. Individuals were captured manually during the day or using mist nets at dusk. Non-lethal tissue sampling was performed on live-captured individuals using biopsy punches on non-vascular areas of the wing membrane (plagiopatagium), yielding tissue fragments 2–4 mm in diameter. Samples were preserved in 90% ethanol and transported to the laboratory. Additionally, morphological traits were evaluated to confirm species identity, including forearm length (FA), third and fifth finger lengths (D3, D5), and wing membrane patterns, following the criteria of [34] (Figure 1 and Figure 2). For all captured individuals, both wings were examined to assess venation patterns used as a rapid field diagnostic tool for distinguishing closely related *Pipistrellus* species. Other external features, such as facial or inguinal coloration, were not assessed, and echolocation parameters were not recorded, as individuals were released immediately after tissue sampling.

### 2.2. Sampling

**Figure 3:**
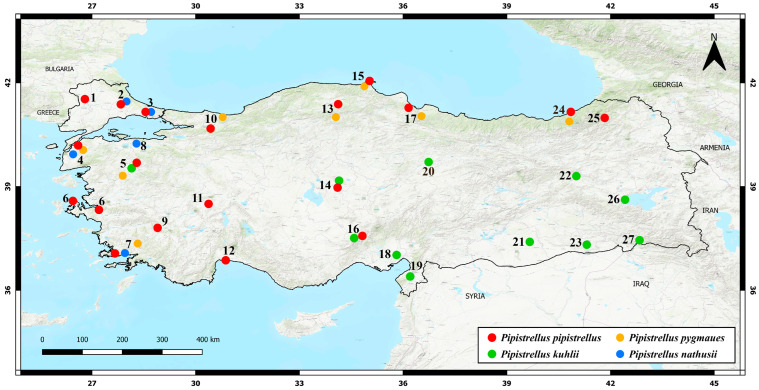
Geographic distribution of sampled *Pipistrellus* species in Türkiye based on fieldwork conducted between 2019 and 2025. Locality numbers correspond to the specimen information provided in Table S1.

This study utilized original samples collected through extensive fieldwork in Türkiye alongside additional mitochondrial *Cytb* sequences retrieved from the NCBI GenBank database. Detailed metadata are provided in Supplementary File Table S1, which includes species identifications, sampling localities, voucher numbers, GenBank accession numbers, sample origins, and source publications. In total, individuals from four *Pipistrellus* species were analyzed across 26 localities in Türkiye (Table S1; Figure 3). The final dataset comprises 78 original *P. pipistrellus* individuals, 45 *P. kuhlii*, 11 *P. nathusii*, and 22 *P. pygmaeus*.

### 2.3. DNA Extraction and Polymerase Chain Reaction (PCR)

Tissue samples (2–4 mm) were obtained from the plagiopatagium (wing membranes) using sterile biopsy punches. DNA extraction was performed using the GeneMATRIX Tissue DNA Purification Kit (EURx, Gdańsk, Poland) according to the manufacturer’s protocol. DNA quality was first assessed qualitatively via 1% agarose gel electrophoresis. Quantitative evaluation of concentration and purity was conducted using a NanoDrop spectrophotometer (Thermo Fisher Scientific, Waltham, MA, USA) at absorbance wavelengths of 260 nm and 280 nm. The A260/A280 ratio was used to assess protein impurities; values near 1.8 indicated high-purity DNA. Extracted DNA was resuspended in TE buffer (10 mM Tris-HCl, 1 mM EDTA, pH 8.0) and stored at −20 °C until PCR amplification.

Amplification of the mitochondrial cytochrome *b* (Cyt*b*) gene utilized the primer pair L14724a (5′ CGAAGCTTGATATGAAAAACCATCGTTG-3′) and H15915R (5′ GGAATTCATCTCTCCGGTTTACAAGAC-3′) [35]. PCR conditions were optimized to amplify the entire Cyt*b* region as a single fragment. PCR reagent concentrations followed the protocol described by Çolak et al. [36]. The thermal cycling protocol began with an initial denaturation step at 94 °C for 5 min. This was followed by 35 cycles, each consisting of denaturation at 94 °C for 1 min, annealing at 43 °C for 1 min, and extension at 65 °C for 5 min. A final extension at 65 °C for 5 min was performed to ensure complete synthesis of all fragments. All successfully amplified PCR products were sequenced bidirectionally using the Sanger chain termination method with gene-specific primers.

### 2.4. Phylogenetic Reconstruction and Population Analyses

Chromatogram files were visualized using FINCHTV v1.4 software, exported in FASTA format, and subsequently aligned using BIOEDIT v7.2.5.9 [37]. Population genetic parameters were computed using DNASP v6.12.03 [38]. These parameters included the number of segregating sites (S), number of haplotypes (H), haplotype diversity (h), nucleotide diversity (π), and gene flow (Nm) between intraspecific groups. Relationships among haplotypes were visualized using median-joining networks constructed in NETWORK v10.1.0 [39]. To assess the demographic history and neutrality, Tajima’s D [40] and Fu’s Fs [41] tests were conducted using ARLEQUIN v3.5 [42]. Mismatch distribution analyses were also performed to detect signals of demographic expansion or stability, estimating demographic parameters (e.g., τ) under the sudden expansion model. Theoretical Poisson-based distributions were computed and compared with observed distributions via custom Python scripts (Python version 3.10). Phylogenetic relationships were reconstructed using the Maximum Likelihood (ML) method implemented in IQ-TREE v1.6 [43]. The best-fitting substitution model was selected based on the Bayesian Information Criterion (BIC) using MEGA v11. Genetic distances among major lineages were calculated under the Kimura 2-parameter (K2P) model.

### 2.5. Divergence Time Estimations

Divergence times were inferred from the mitochondrial *Cytb* alignment using BEAST v1.10.4 [44] under a strict molecular clock. The dataset was partitioned by codon position (1st, 2nd, 3rd). Each partition was modeled with HKY+G and empirical base frequencies; substitution and among-site rate parameters were unlinked across partitions. The clock rate was estimated from the data, conditional on fossil constraints. Node ages were calibrated with three fossil-informed MRCA priors, following Borges et al.: Crown Vespertilionidae (lognormal prior; monophyly enforced for all sampled vespertilionids), the Stem *Vespertilio-Pipistrellus* split (lognormal prior; monophyly enforced for *Vespertilio murinus* and *Pipistrellus* spp.), and Crown *Myotis* (lognormal prior; monophyly enforced for all sampled *Myotis*) [45]. The offset and lognormal shape parameters for each calibration were set exactly as reported by Borges et al. [45]. A Yule speciation prior was used for the tree. One MCMC chain was run for 50 million generations, sampling every 10,000 steps; the first 10% was discarded as burn-in. Convergence and mixing were assessed in Tracer (ESS > 200 for all key parameters) [46]. Finally, the maximum clade credibility (MCC) tree with median node heights and 95% HPD intervals was summarized in TreeAnnotator and visualized.

## 3. Results

### 3.1. Sampling Localities

A total of 187 *Pipistrellus pipistrellus* specimens collected from 19 localities across Türkiye (Figure 3) were analyzed to assess the species’ genetic structure. These individuals yielded 970 bp fragments of the mitochondrial cytochrome *b* (Cyt*b*) gene. The original dataset was then expanded with 62 additional sequences retrieved from the NCBI GenBank database. Phylogenetic rooting was achieved using one *P. nathusii* individual from the present study (Accession No. PV951912) and one *Myotis myotis* sequence from GenBank (Accession No. AF376860). In total, 142 *P. pipistrellus* sequences and two outgroups were included, enabling a comprehensive evaluation of genetic diversity and haplotype composition (Table S1). The genetic diversity and phylogenetic relationships of *Pipistrellus kuhlii* were evaluated using both field-collected specimens and sequences from GenBank. A total of 45 individuals sampled from 10 localities across Türkiye yielded Cyt*b* fragments ranging from 1008 to 1113 bp. We incorporated an additional 41 *P. kuhlii* Cyt*b* sequences from GenBank, and the phylogenetic tree was rooted using one *P. pipistrellus* specimen from this study (Accession No. PV951922) and one *M. myotis* sequence (Accession No. AF376860). Analyses of *Pipistrellus nathusii* included 11 original specimens collected from five localities in Türkiye; each produced 1009 bp Cyt*b* sequences. Six European reference sequences from GenBank were also incorporated to place the Turkish samples within a broader phylogenetic context. One *P. pipistrellus* individual from this study (Accession No. PV951922) and one *M. myotis* sequence (Accession No. AF376860) were used as outgroups for rooting. Twenty-two individuals morphologically identified as *Pipistrellus pygmaeus* were sampled from nine localities across Türkiye (Muğla, Çanakkale, Edirne, Sakarya, Kastamonu, Rize, Samsun, Sinop, and Balıkesir) (Table S1). Nineteen Cyt*b* sequences labeled as *P. pygmaeus* were retrieved from GenBank, representing populations from Azerbaijan, Iran, Greece, Spain, Switzerland, Portugal, the Czech Republic, Cyprus, and Türkiye, and were also included in the analysis. One *P. pipistrellus* specimen from this study (Accession No. PV951922) and one *M. myotis* sequence (Accession No. AF376860) served as outgroups.

### 3.2. Phylogenetic Relationships, Genetic Distances, and Gene Flow

**Figure 4:**
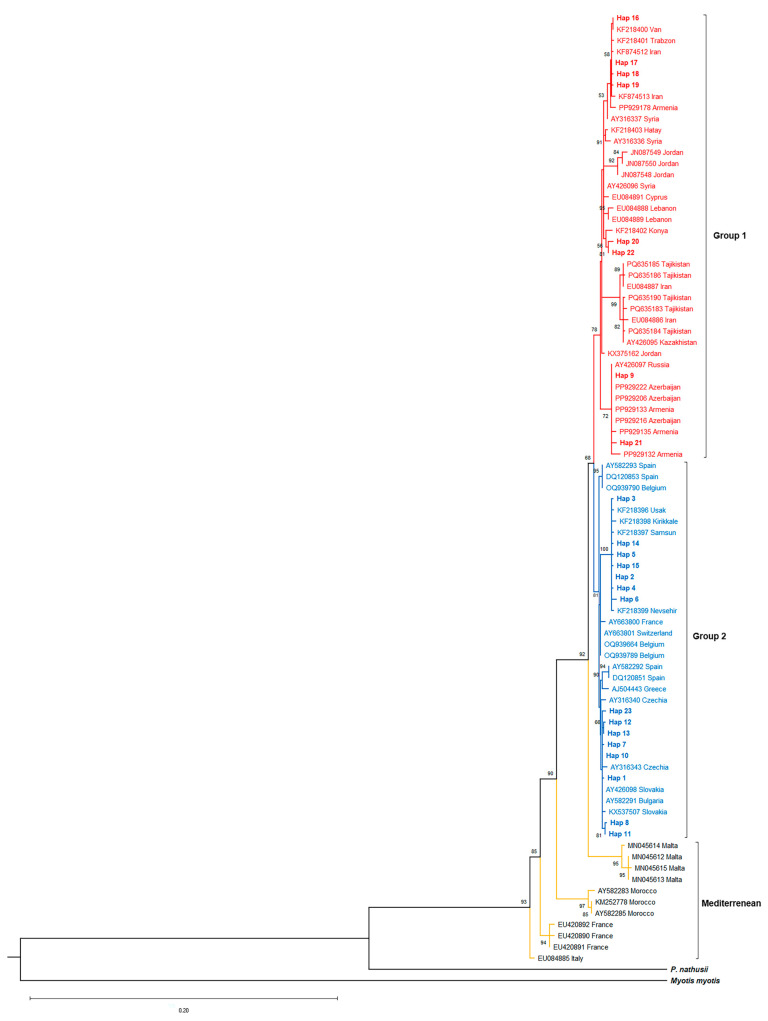
Maximum Likelihood (ML) phylogenetic tree of *Pipistrellus pipistrellus* based on Cyt*b* sequences. Bootstrap support values (>50%) are shown at the nodes.

Maximum Likelihood (ML) analyses of *P. pipistrellus*, which utilized the HKY+G substitution model (best-fit according to the Bayesian Information Criterion, BIC), resolved three major intraspecific lineages (Figure 4). Group 1 encompassed specimens from eastern and central Türkiye, clustering with sequences from Cyprus, the Middle East, the Caucasus, and Central Asia. Group 2 included individuals from western and northwestern Türkiye (a wide range of 20 provinces) and sequences from Europe and the Balkans. The Mediterranean group included multiple distinct lineages comprising specimens from Morocco, Sicily, France, and Malta. Bootstrap support for the split between Group 1 and Group 2 was 68%, while the Mediterranean group showed robust phylogenetic separation (93%). Pairwise genetic distances (Kimura-2-parameter, K2P) further supported this structure: mean divergences were 2.2% ± 0.40 (Group 1 vs. Group 2), 4.5% ± 0.71 (Group 1 vs. Mediterranean), and 4.7% ± 0.72 (Group 2 vs. Mediterranean) (Table S2). Gene flow estimates [47] indicated very limited migration between these lineages: *Nm* = 0.16 (Group 1 vs. Group 2), Nm = 0.08 (Group 1 vs. Mediterranean), and Nm = 0.12 (Group 2 vs. Mediterranean). All Nm values fell below the isolation threshold (Nm < 1), confirming ongoing genetic isolation.

**Figure 5:**
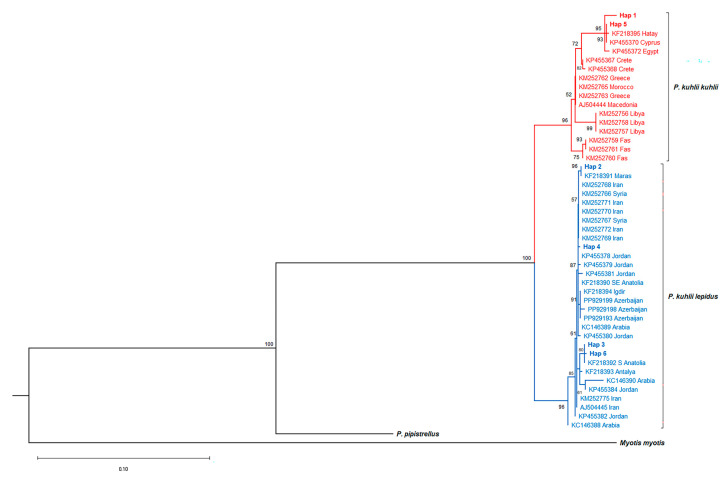
Maximum Likelihood (ML) phylogenetic tree of *Pipistrellus kuhlii* based on Cyt*b* sequences. Bootstrap support values (>50%) are shown at the nodes.

The *P. k. lepidus* clade, separated from *P. k. kuhlii* with strong support (100% bootstrap), included specimens collected from 10 Turkish provinces (Figure 5). Conversely, the *P. k. kuhlii* clade encompassed individuals from Türkiye (Kırşehir, Hatay, and Adana) alongside populations from North Africa, southeastern Europe, and Mediterranean islands. This pattern reflects the hypothesized eastern Mediterranean and Levantine distribution of the latter lineage. Notably, specimens from Kırşehir, Hatay, and Adana were represented in both clades. Pairwise genetic distance estimates based on the Kimura 2-parameter (K2P) model indicated a mean divergence of 3.95% ± 0.76 between *P. k. lepidus* and *P. k. kuhlii*. Additionally, gene flow was found to be limited (Nm = 0.35) (Table S2). This genetic structuring was evident despite the collection of some specimens within the same localities (Kırşehir, Hatay, and Adana) that were assigned to both clades.

**Figure 6:**
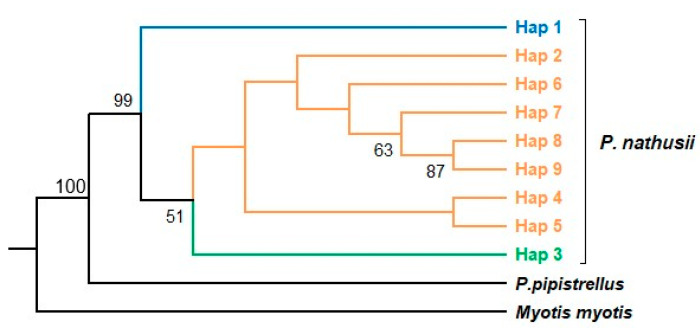
Maximum Likelihood (ML) phylogenetic tree of *Pipistrellus nathusii* based on Cyt*b* sequences. Bootstrap support values (>50%) are shown at the nodes.

The phylogenetic relationships of *Pipistrellus nathusii* were evaluated using the Maximum Likelihood (ML) approach, with HKY+G identified as the best-fitting substitution model. Based on 11 original Turkish samples and six European sequences obtained from GenBank, the ML tree revealed a distinct structuring within the species (Supplementary File Table S1, Figure 6). The first lineage consisted solely of a Muğla specimen (Haplotype 1) that clustered separately with 99% bootstrap support. The second lineage grouped individuals from Çanakkale, Bursa, Tekirdağ, and İstanbul with GenBank sequences from Europe (Haplotypes 2 and 4–9). The third lineage comprised a single haplotype (Haplotype 3) from Bursa that clustered weakly (BS = 51%) with the second group. K2P genetic distances between the groups were low but consistent: 0.33% ± 0.16 (Group 1 vs. Group 2), 0.29% ± 0.17 (Group 1 vs. Group 3), and 0.22% ± 0.11 (Group 2 vs. Group 3) (Table S2). Gene flow estimates could not be calculated for Groups 1 and 3 due to the single-specimen representation.

All 22 Turkish individuals morphologically identified as *P. pygmaeus* matched *P. pipistrellus* in BLAST searches (BLAST version 2.16.0). Subsequent HKY+G-based ML analyses confirmed this finding, as these Turkish specimens clustered within the *P. pipistrellus* clade (Figure S1), indicating that morphological identification alone—particularly wing venation—can be misleading for these cryptic taxa [34]. Sequences labeled *P. pygmaeus* from GenBank (including Turkish specimens AY426087 and AY316328) formed a separate, distinct clade, confirming that our Turkish samples do not belong to the *P. pygmaeus* taxon.

### 3.3. Mismatch Distribution and Neutrality Tests

**Figure 7:**
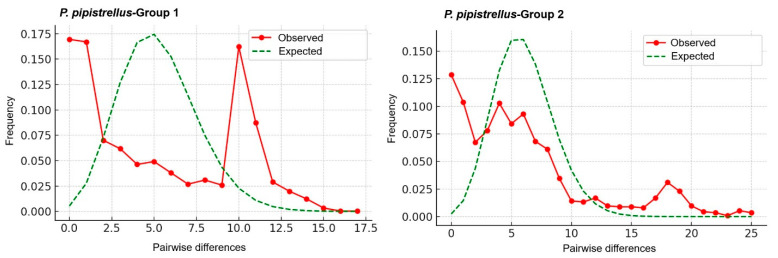
Mismatch distribution curves of *Pipistrellus pipistrellus* for Group 1 and Group 2, based on Cyt*b* sequences. Observed frequency distributions (solid lines) are compared with the expected distributions under the sudden expansion model (dashed lines).

Mismatch distribution analyses and neutrality tests were performed to assess the demographic history of *P. pipistrellus* (Table S2). Group 1 (*n* = 81) comprised 32 haplotypes (S = 41), showing moderate haplotype diversity (h = 0.846) and low nucleotide diversity (π = 0.005). Neutrality tests yielded a negative Tajima’s D (−1.170, not significant) but a significantly negative Fu’s Fs (−16.766, *p* < 0.02). The mismatch distribution exhibited a multimodal pattern (Figure 7), showing deviation from the expectations of a simple sudden expansion model. Group 2 (*n* = 48) contained very high haplotype diversity (h = 0.982) and nucleotide diversity (π = 0.006), similar to Group 1, with 64 segregating sites observed. Both Tajima’s D (−0.834) and Fu’s Fs (4.646) were non-significant. The mismatch distribution was highly irregular and multimodal (Figure 7).

**Figure 8:**
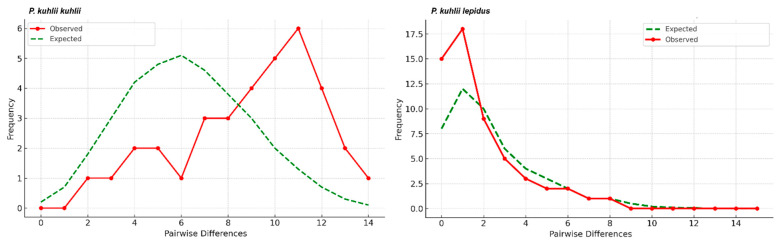
Mismatch distribution plots for *P. kuhlii kuhlii* and *P. kuhlii lepidus* based on Cyt*b* sequences. Red dotted lines represent the observed distributions, and green dashed lines show the expected distributions under the sudden expansion model.

**Figure 9:**
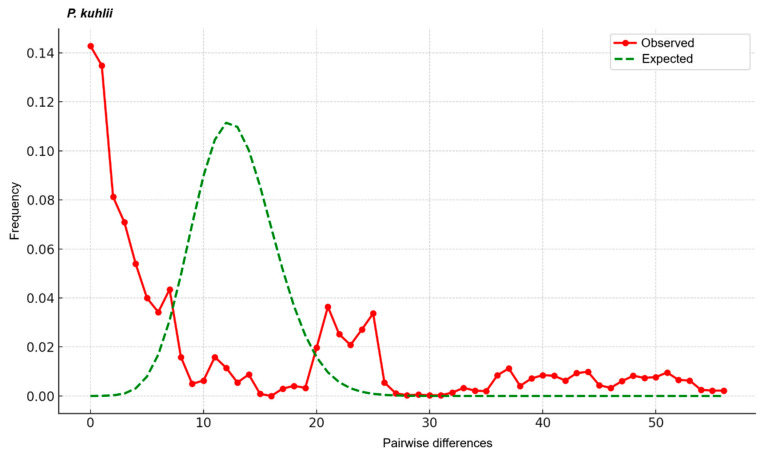
Mismatch distribution for *Pipistrellus kuhlii* based on all sampled individuals. The observed frequencies (red line) are compared to the expected distribution under a sudden population expansion model (green dashed line).

In *P. k. kuhlii* (*n* = 29), six haplotypes were detected (h = 0.835; π = 0.0305). Both Tajima’s D (1.254) and Fu’s Fs (2.888) were positive but non-significant. The mismatch curve was multimodal and skewed (Figure 8). Conversely, in *P. k. lepidus* (*n* = 57), seven haplotypes were detected (h = 0.396; π = 0.006). Both Tajima’s D (−1.850, *p* < 0.05) and Fu’s Fs (−2.042, *p* < 0.05) were significantly negative. The mismatch curve broadly aligned with the sudden expansion model, though it exhibited slight undulations and asymmetry (Figure 8). When all *P. kuhlii* individuals were pooled (*n* = 86), the overall mismatch pattern (Figure 9) showed a divergence from the simple sudden expansion model. This pattern was characterized by high frequencies at low pairwise differences, followed by a multimodal distribution.

**Figure 10:**
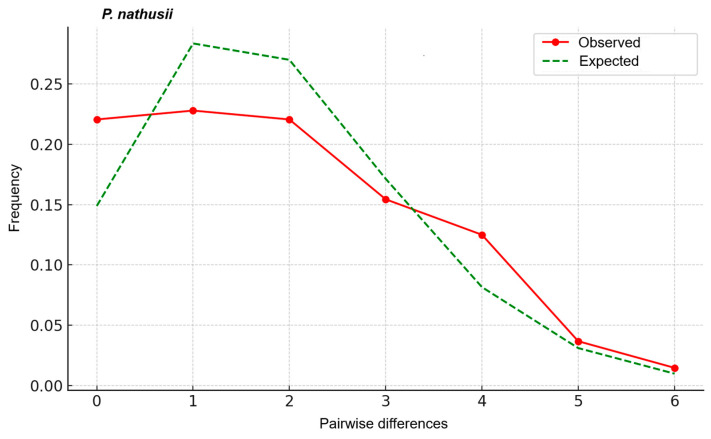
Mismatch distribution of *Pipistrellus nathusii* based on Cyt*b* sequences from Turkish populations. The observed distribution of pairwise differences (bars) is compared with the expected distribution under the sudden expansion model (solid line).

Analyses of *P. nathusii* (*n* = 11) identified nine haplotypes, yielding high haplotype diversity (h = 0.73) and low nucleotide diversity (π = 0.002), with 11 segregating sites found. Tajima’s D was significantly negative (−2.152, *p* < 0.05), whereas Fu’s Fs was positive (13.596). The mismatch distribution (Figure 10) was slightly asymmetric and right-skewed, exhibiting a mild deviation from a Poisson-like sudden expansion curve.

### 3.4. Median-Joining Network Analysis

**Figure 11:**
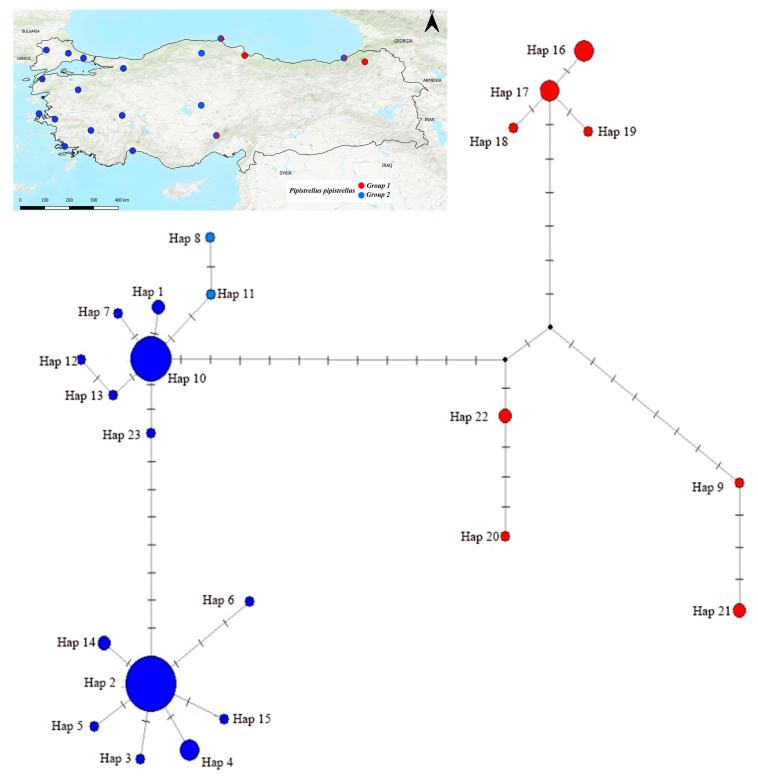
Median-joining network of *Pipistrellus pipistrellus* haplotypes sampled from Türkiye. Each circle represents a distinct haplotype, with circle size proportional to haplotype frequency. Colors correspond to phylogenetic groups inferred from the ML tree: Group 1 (red) and Group 2 (blue). Lines represent mutational steps between haplotypes.

A median-joining network (MJN) was constructed using the Cyt*b* haplotypes to visualize intraspecific genetic structuring in *P. pipistrellus* (Table S1, Figure 11). The network topology was compared with the ML phylogeny. The network revealed both widespread and highly localized haplotypes. Haplotype 2 (Hap 2) was widely distributed across numerous localities (including Çanakkale, Muğla, İzmir, Niğde, Rize, and Sinop) and occupied a central position. In contrast, Haplotype 9 (Hap 9) was recorded from a similarly broad range (Edirne, Antalya, Kastamonu, Sakarya, and Sinop) but remained peripheral. Notably, individuals from provinces such as Sinop (Hap 2, 10, 20, 21), Rize (Hap 2, 15–18), and Niğde (Hap 2, 22) were present in both major phylogenetic groups (Group 1 and Group 2).

**Figure 12:**
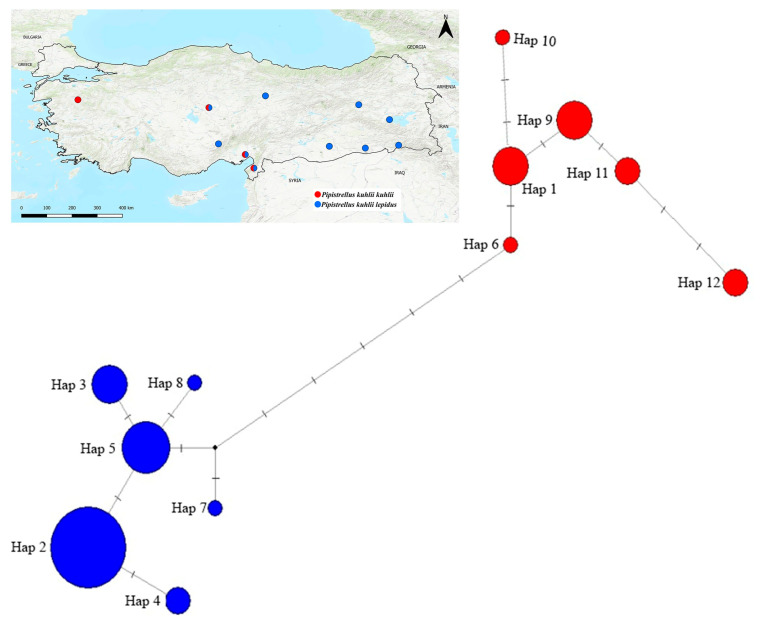
Median-joining network of *Pipistrellus kuhlii* based on Cyt*b* haplotypes. Each circle represents a distinct haplotype, with circle size proportional to haplotype frequency. Colors indicate phylogenetic assignment: *P. k. lepidus* (blue) and *P. k. kuhlii* (red). Lines connecting haplotypes represent mutational steps. Locality codes and haplotype distributions are detailed in Supplementary Table S1.

The MJN analysis for *P. kuhlii* incorporated 45 Turkish individuals and GenBank sequences, resulting in a total of twelve haplotypes (Figure 12; Supplementary File Table S1). Haplotype 2 (Hap 2) was the most frequent and widely distributed, found across numerous Turkish localities (e.g., Adana, Hatay, Mardin, Niğde) and several Middle Eastern countries. Its widespread occurrence was associated with a central placement in the network. Other *P. k. lepidus* haplotypes (Hap 3, 4, 7, 8) had restricted geographic ranges and peripheral positions. The *P. k. kuhlii* lineage was primarily represented by Hap 1 (Adana, Hatay, Kırşehir, and Cyprus), Hap 6 (Kırşehir), and Hap 10 (Egypt). Additional haplotypes (Hap 9, 11, 12) were confined to North Africa and southeastern Europe, appearing at the network periphery. The haplotype network supported the phylogenetic structure recovered by the ML analyses.

**Figure 13:**
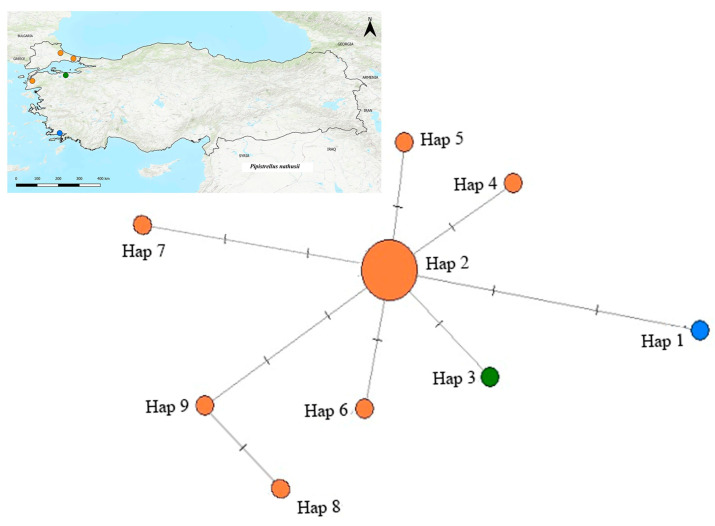
Median-joining network of *Pipistrellus nathusii* based on Cyt*b* haplotypes. Each circle represents a distinct haplotype, with circle sizes proportional to haplotype frequency.

For *P. nathusii*, the MJN analysis used Turkish specimens and European GenBank sequences, revealing nine haplotypes and their geographic relationships (Figure 13). Haplotype 2 (Hap 2) formed the network core, encompassing individuals from Çanakkale, Tekirdağ, Bursa, and İstanbul, along with samples from Spain, Switzerland, and Belgium. Conversely, the Muğla haplotype (Hap 1) was distinctly positioned on the periphery with minimal connections. The Bursa haplotype (Hap 3) was also rare and distinct. Other haplotypes (Hap 4–9) branched directly around Hap 2, linking Turkish and European sequences. This network structure showed extensive genetic continuity across the species’ range.

### 3.5. Molecular Dating and Divergence Times

**Figure 14:**
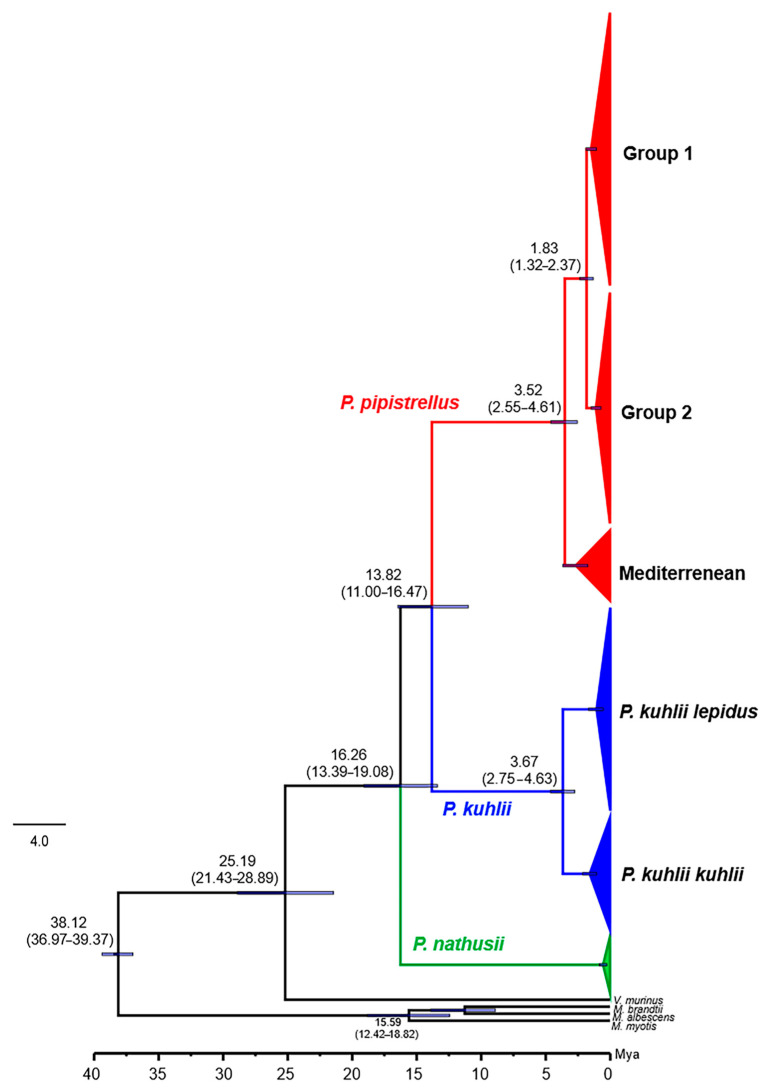
Time-calibrated phylogeny of *Pipistrellus* species based on *cytochrome b* sequences. Median divergence times (Mya) are shown at major nodes, with 95% highest posterior density (HPD) intervals in parentheses. Node bars represent 95% HPD intervals. Lineages are color-coded: *P. pipistrellus* (red), *P. kuhlii* (blue), and *P. nathusii* (green). Outgroups included *Myotis brandtii*, *M. albescens*, *M. myotis*, and *Vespertilio murinus*. Fossil-calibrated priors were set following Borges et al. [45].

Fossil-calibrated divergence-time estimates inferred using BEAST provided a chronological framework for the evolution of the sampled taxa (Figure 14). The oldest split observed was the divergence of *Myotis* from the remaining vespertilionids at 38.12 Ma (95% HPD 36.97–39.37). This was followed by the separation of the *Vespertilio* and *Pipistrellus* lineages at 25.19 Ma (95% HPD 21.43–28.89). Within the genus *Pipistrellus*, the divergence of *P. nathusii* from the (*P. pipistrellus* + *P. kuhlii*) clade occurred at 16.26 Ma (95% HPD 13.39–19.08). Subsequently, *P. pipistrellus* and *P. kuhlii* split at 13.82 Ma (95% HPD 11.00–16.47). Intraspecific divergences were significantly shallower, dating to the Pliocene and Pleistocene eras. The Mediterranean group of *P. pipistrellus* diverged from Groups 1 and 2 at 3.52 Ma (95% HPD 2.55–4.61). The subsequent split between Group 1 and Group 2 occurred during the Pleistocene, at 1.83 Ma (95% HPD 1.32–2.37). A comparable divergence time was found in *P. kuhlii*, where the separation between *P. k. kuhlii* and *P. k. lepidus* was estimated at 3.67 Ma (95% HPD 2.75–4.63). The most recent internal divergence was observed within *P. nathusii*, with the deepest split dating to a very recent period, between 0.12 and 0.55 Ma.

## 4. Discussion

### 4.1. Resolving Cryptic Species Boundaries

The initial step of our research involved species identification based on morphological criteria and wing venation patterns [34]. However, our results confirmed the challenges repeatedly emphasized in earlier studies [9,48], where cryptic taxa such as *P. pipistrellus* and *P. pygmaeus* are morphologically similar, making external traits alone unreliable. Specifically, 22 individuals initially identified as *P. pygmaeus* based on external morphological traits, particularly wing venation, were subsequently found to cluster robustly within the *P. pipistrellus* clade via Cyt*b* sequence analysis (Figure S1). This substantial misidentification underscores that morphological characters commonly used for identification substantially overlap between these two cryptic species. The problem is not unique to Türkiye; earlier large-scale studies [33] concluded that despite being genetically well differentiated, *P. pipistrellus* and *P. pygmaeus* are morphologically highly overlapping, rendering diagnosis unreliable. While the presence of *P. pygmaeus* in Türkiye has been confirmed genetically [11,12], its distribution may be restricted to Thrace and Western Anatolia. Our findings reiterate that molecular confirmation is essential for accurate species identification of cryptic pipistrelle bats in biogeographic transition zones like Anatolia. Nevertheless, the possibility of limited mitochondrial introgression between *P. pipistrellus* and *P. pygmaeus* in sympatric regions, as reported in Europe [49], cannot be completely ruled out. Future analyses incorporating nuclear markers would therefore be valuable for confirming the absence or extent of hybridization in Anatolian populations.

### 4.2. The Biogeographic Role of Anatolia in P. pipistrellus Diversification and Demographic History

Our comparison of Turkish *P. pipistrellus* sequences with GenBank data revealed three major phylogenetic clusters: Group 1 (East), Group 2 (West), and a paraphyletic Mediterranean group. This bipartite structure is partly consistent with the historical subspecific framework proposed by Albayrak (*P. p. pipistrellus* [West] and *P. p. aladdin* [East]) [13]. However, our molecular results placed Hatay within the eastern group (Group 1), and Mediterranean specimens (corresponding to *P. p. mediterraneus*) were entirely absent from our Turkish dataset, suggesting the presence of this subspecies in Türkiye requires molecular re-evaluation. The co-occurrence of both the Western (Group 2) and Eastern (Group 1) clades in several Turkish localities, including Niğde, Sinop, Samsun, and Rize, indicates significant distributional overlap and confirms the existence of secondary contact zones. This pattern is highly consistent with the refugial and contact zone hypotheses proposed for the Mediterranean Basin [2,23]. The Anatolian Diagonal appears to function as a semi-permeable barrier; while it promotes pronounced genetic divergence in many taxa [50,51], its influence on volant species like *Pipistrellus* is less restrictive. The bat’s capacity for long-distance movements and use of natural corridors [52] likely explains the co-occurrence of both lineages in transitional areas along the Diagonal. The mixture of European- and Asian-derived haplotypes corroborates the view that Türkiye acts as a crucial biogeographic bridge and a center of mitochondrial diversity [2].

The Median-Joining Network (MJN) analysis revealed the relationships among *P. pipistrellus* haplotypes (Figure 6). Although the central and widespread position of Haplotype 2 (Hap 2) in the network suggests that it may represent a common and possibly ancestral haplotype, its placement within the broader phylogeny (Figure 4) indicates that this pattern likely reflects local relationships rather than a truly basal position. In addition, the peripheral position of Haplotype 9 suggests a geographically restricted lineage that may have persisted regionally over time. The MJN topology, combined with the co-occurrence of both major phylogenetic groups in provinces such as Sinop, Rize, and Niğde, further reinforces the hypothesis that these localities may represent historical contact areas. The MJN topology, combined with the co-occurrence of both major phylogenetic groups in provinces such as Sinop, Rize, and Niğde, further reinforces the hypothesis that these localities may represent historical contact areas.

Mismatch distribution analyses support this complex history. For Group 1, the significantly negative Fu’s Fs value (−16.766) suggests a historical population expansion, yet the observed multimodal mismatch distribution (Figure 7) is inconsistent with a simple sudden expansion model. This conflict implies that Group 1 has experienced complex demographic processes involving either subsequent genetic structuring or expansion from multiple sub-lineages. Furthermore, the non-significant neutrality tests and highly irregular, multimodal mismatch distribution for Group 2 support complex dynamics, potentially involving admixture between divergent lineages or long-term demographic stability, indicating that this group harbors a heterogeneous genetic background.

### 4.3. Lineage Diversification and Secondary Contact in P. kuhlii

Analyses of mitochondrial Cyt*b* data resolved two major phylogenetic lineages within Turkish *Pipistrellus kuhlii*, corresponding to *P. k. lepidus* and *P. k. kuhlii*. In line with the subspecific framework of Andriollo et al. [53], Turkish populations clustered into these two groups, indicating that Türkiye is not merely part of a continuous range but a biogeographic transition zone where divergent lineages meet and overlap. This observed split largely corroborates morphologically defined intraspecific differentiation previously described for the species [34,54] and aligns with the Mediterranean contact zones discussed by Stadelmann et al. [24].

The mean K2P genetic divergence of 3.95% ± 0.76 between these lineages exceeds typical intraspecific thresholds in mammals [55], providing strong molecular support for the long-term isolation of these units, potentially warranting subspecies or even species status.

Within *P. kuhlii*, the *kuhlii* and *lepidus* lineages form a well-supported split in the ML tree (BS ≈ 93%) and show higher pairwise divergence (K2P ≈ 3.95%) than observed among *P. pipistrellus* groups. By contrast, the median-joining network displays relatively short connections between its haplogroups. This difference is likely because parsimony-based networks show only the minimum number of mutations between haplotypes and do not reflect actual genetic distances, which are calculated in model-based analyses like ML or K2P [39,56]. The apparent discrepancy between percentage divergence and the number of mutational steps also reflects methodological and dataset-related factors. K2P distances account for transition/transversion bias, multiple substitutions [57], and alignment length differences (970 bp in *P. pipistrellus* vs. up to 1113 bp in *P. kuhlii*), whereas median-joining networks represent only the minimum number of observed substitutions. In addition to methodological factors, this discrepancy may also reflect lineage-specific mitochondrial variation. Similar patterns have been observed in vespertilionid bats [58], where incomplete lineage sorting (ILS) was suggested as a possible cause. Biologically, the pattern is compatible with long-term mitochondrial isolation followed by secondary contact. The overall topology of the network (Figure 12) shows several rare haplotypes radiating from a central, widespread haplotype (Haplotype 2), a pattern that may indicate recent diversification within the population. The co-occurrence of both lineages in Kırşehir, Hatay, and Adana points to secondary contact zones-likely microrefugia where lineage diversity persisted during glacial periods. Furthermore, our phylogenetic analysis placed GenBank sequences labeled as *P. k. deserti* within the *P. k. kuhlii* lineage, supporting the conclusion [59] that the *deserti* form is an environmentally induced ecomorphotype rather than a distinct evolutionary lineage.

Mismatch distribution analyses revealed contrasting demographic signals between the lineages. *P. k. lepidus* showed significantly negative Tajima’s D and Fu’s Fs values, and a mismatch curve that broadly aligned with the sudden expansion model. This result provides strong statistical evidence for a past population expansion in the eastern lineage. Conversely, *P. k. kuhlii* showed positive and non-significant neutrality indices and a multimodal curve, suggesting long-term stability or mild contraction. The complex, multimodal pattern observed when all individuals were pooled (Figure 9) is likely a direct reflection of the co-existence of these two deeply divergent lineages, masking the simpler expansion signal found within *P. k. lepidus*.

### 4.4. Phylogeography of the Migratory P. nathusii

Mitochondrial Cyt*b* data revealed that most Turkish populations of *P. nathusii*, particularly those from Northwestern Anatolia and Thrace, cluster within the European lineage. The MJN analysis supports this, showing that Haplotype 2 (Hap 2) forms the network core and links Turkish individuals with samples from across Europe (Figure 13). This robust genetic continuum with Europe is highly consistent with the documented migratory nature of the species [60].

A notable exception was the specimen from Muğla (Southwestern Turkey, Hap 1), which formed a distinct and well-supported clade in the ML tree (BS = 99%). In the MJN (Figure 13), this haplotype shows limited mutational separation from the central haplotype, suggesting slight local differentiation rather than a deeply isolated lineage. This pattern may reflect regional persistence of genetic variation in Southwestern Anatolia, consistent with the idea that local refugia in Western Asia preserved micro-scale diversity during glacial cycles [28].

The significantly negative Tajima’s D value observed for this species further suggests a recent population expansion. However, the positive Fu’s Fs (13.596) value may reflect selective processes or pronounced population structure, which is consistent with the presence of the differentiated Muğla lineage. The slightly asymmetric mismatch distribution tentatively suggests demographic stabilization following an earlier expansion or expansion originating from multiple sublineages.

### 4.5. Temporal Framework for Diversification: Miocene-Pleistocene Influence

The fossil calibrated divergence time estimates recovered in this study are broadly congruent with earlier molecular dating frameworks for vespertilionid bats [24,45,61,62]. However, it should be noted that estimates based solely on mitochondrial data may carry systematic bias, particularly for deeper nodes, as discussed in previous studies [63,64].

The split of Myotis from other vespertilionids at ~38 Ma corresponds to the late Eocene, a period marked by global cooling and faunal turnover, which has previously been implicated as a driver of diversification in Chiroptera [4,45]. Similarly, the separation of *Vespertilio* and *Pipistrellus* around 25 Ma falls within the Oligocene-Miocene transition, a period marked by major climatic reorganizations including global cooling, increased seasonality, and the expansion of open habitats across Eurasia [62,65,66]. This divergence timing is also consistent with recent phylogenomic frameworks, suggesting that the early Miocene split between the *Vespertilio* and *Pipistrellus*–*Nyctalus* lineages occurred approximately 25 Mya [45,67], supported by fossil evidence of early *Nyctalus* species [68], although genomic calibrations [69] provide more robust estimates for higher-level splits.

Within *Pipistrellus*, the divergence of *P. nathusii* from the *P. pipistrellus* and *P. kuhlii* clade at ~16 Ma coincides with early-middle Miocene climatic oscillations. This phylogenetic pattern is largely consistent with the mitochondrial topology reported by Zhukova et al. [67], who also recovered *P. nathusii* as an early-diverging lineage relative to the (*P. pipistrellus* + *P. kuhlii*) clade, while additionally including *P. pygmaeus* as a sister taxon to *P. pipistrellus*. Minor topological differences likely reflect variation in taxon sampling and the inclusion of additional European lineages in their dataset. Subsequently, the split between *P. pipistrellus* and *P. kuhlii* at approximately 14 Ma aligns with the Middle Miocene Climatic Transition (MMCT, ~14–13 Ma), when global cooling and Antarctic glaciation reshaped ecosystems, leading to the emergence of more temperate and arid habitats [70,71]. Similar timing has been inferred for the early diversification of *Myotis* [24], when global climatic transitions fostered habitat expansion and biogeographic radiation across Eurasia. These concordant patterns suggest that Miocene climatic reorganizations broadly facilitated lineage diversification within Vespertilionidae.

In *P. pipistrellus*, divergence dating revealed two continental lineages, including Turkish populations, that split around 1.8 Ma. The Mediterranean group diverged slightly earlier, about 3.5 Ma. Hulva et al. [23] suggested that diversification within the *P. pipistrellus* complex was driven by environmental fragmentation during the Messinian Salinity Crisis (5.96–5.33 Ma), together with ecological specialization of Mediterranean populations. Our divergence times are more recent than the MSC. Instead, Late Pliocene climatic oscillations and sea-level fluctuations, which repeatedly changed habitat connectivity in the Mediterranean Basin, likely played a stronger role in shaping the observed patterns of lineage differentiation. Finally, the shallow divergence within *P. nathusii* (0.12–0.55 Ma) aligns with late Pleistocene climatic cycles, consistent with its documented migratory ecology and range shifts across Europe and Anatolia [30,59].

Overall, these results suggest that climatic fluctuations since the Miocene, including the Messinian Salinity Crisis, promoted early lineage divergence and regional isolation within *Pipistrellus*. Later Pliocene–Pleistocene climatic oscillations likely enhanced secondary contact and range shifts among these lineages. Together, these events played a major role in shaping the current diversification patterns of *Pipistrellus*, although mitochondrial-based dating should be interpreted with caution due to potential calibration bias.

## 5. Conclusions

This study confirms Anatolia as a crucial evolutionary center for the *Pipistrellus* genus across the Western Palearctic. Our findings reveal deep intraspecific divergence within multiple species, establishing Türkiye as a significant secondary contact zone where historically isolated lineages converge and overlap, a pattern clearly observed in both *P. pipistrellus* and *P. kuhlii*. The discovery of a distinct, potentially resident lineage of *P. nathusii* alongside widespread migratory populations highlights Anatolia’s dual role as both a refugium (preserving unique diversity) and a migratory corridor (facilitating gene flow). Furthermore, the complete misidentification of all *P. pygmaeus* specimens based on morphology definitively confirms that external traits are unreliable for distinguishing these cryptic taxa. This underscores the essential and mandatory role of molecular data in modern taxonomy and accurate conservation assessments for the genus. These contemporary genetic patterns are the legacy of diversification events driven by major climatic shifts from the Miocene to the Pleistocene. In summary, the complex phylogeographic structure observed reinforces Anatolia’s position as a dynamic center of evolution, actively shaping the genetic diversity of Palearctic bat fauna.

## Data Availability

All novel DNA sequences have been deposited in the GenBank international repository. The complete list of Accession Numbers corresponding to the samples analyzed in this study is provided within the Supplementary File (Table S1).

## Supplementary Materials

The following supporting information can be downloaded at: https://www.mdpi.com/article/10.3390/biology14111549/s1. The Supplementary File contains all detailed data and figures supporting the phylogenetic, demographic, and biogeographic analyses presented in this article. Table S1: Provides a comprehensive summary of all 156 *Pipistrellus* samples used in this study, including locality data, map numbers, field identification numbers, GenBank accession numbers, assigned haplotypes, and references. Table S2: Genetic divergence (K2P), gene flow (Nm), and neutrality tests for *Pipistrellus* species based on Cyt*b* data. Figure S1: Displays the Maximum Likelihood (ML) phylogeny for Turkish specimens morphologically identified as *P. pygmaeus*, including all related outgroup and reference sequences. References [2,11,23,33,59,72,73,74,75,76,77,78,79,80,81,82,83,84] are cited in the Supplementary Materials.
