# Supplementary material for: Phylogeographic Insights into Pipistrellus Species from Türkiye: Diversity, Divergence, and Regional Lineage Structure
Source: Biology (Basel). 2025 Nov 4;14(11):1549. doi: 10.3390/biology14111549 (PMC12650336; doi:10.3390/biology14111549)
Supplement: Supplementary file 1 [file biology-14-01549-s001.zip › biology-3935220-supplementary.pdf]

Table S1. Summary of *Pipistrellus* samples used in this study, including locality data, map numbers, haplotypes, GenBank accession numbers, and references

Species	Locality	Map number	Sample ID-Accession no	Haplotypes	References
P. pipistrellus	Çanakkale	4	57- PV951922<br>107- PV951928<br>111- PV951929<br>146- PV951934<br>153- PV951935	Hap 1<br>Hap 1<br>Hap 2<br>Hap 2<br>Hap 2	This study
	Denizli	9	80- PV951923<br>91- PV951924<br>95- PV951925<br>133- PV951930	Hap 2<br>Hap 3<br>Hap 2<br>Hap 2	This study
	Muğla	7	97- PV951926<br>98- PV951927	Hap 2<br>Hap 2	This study
	İzmir	6	145- PV951933<br>163- PV951936<br>164- PV951937<br>165- PV951938<br>354- PV951991<br>355- PV951992<br>356- PV951993<br>357- PV951994<br>370- PV951999	Hap 6<br>Hap 7<br>Hap 2<br>Hap 4<br>Hap 2<br>Hap 4<br>Hap 4<br>Hap 2<br>Hap 23	This study
	Kırşehir	14	191- PV951941	Hap 2	This study
	Sakarya	10	255- PV951951<br>256- PV951952<br>257- PV951953<br>258- PV951954	Hap 2<br>Hap 12<br>Hap 13<br>Hap 10	This study
	Kastamonu	13	279-PV951960<br>280- PV951961<br>281- PV951962<br>282- PV951963<br>283- PV951964<br>284- PV951965<br>285- PV951966	Hap 10<br>Hap 10<br>Hap 2<br>Hap 14<br>Hap 2<br>Hap 14<br>Hap 2	This study
	Rize	23	291- PV951967<br>292- PV951968<br>293- PV951969<br>294- PV951970<br>295- PV951971<br>296- PV951972<br>297- PV951973<br>298- PV951974<br>299- PV951975	Hap 2<br>Hap 15<br>Hap 16<br>Hap 16<br>Hap 16<br>Hap 16<br>Hap 2<br>Hap 17<br>Hap 18	This study
	Sinop	15	316- PV951980<br>317- PV951981<br>318- PV951982<br>320- PV951983<br>321- PV951984	Hap 10<br>Hap 20<br>Hap 21<br>Hap 2<br>Hap 21	This study
	Niğde	16	346- PV951985<br>349- PV951986<br>350- PV951987<br>351- PV951988	Hap 22<br>Hap 2<br>Hap 2<br>Hap 2	This study

			352- PV951989<br>353- PV951990	Hap 2<br>Hap 22	
	Balıkesir	5	358- PV951995<br>359- PV951996<br>360- PV951997<br>361- PV951998	Hap 2<br>Hap 2<br>Hap 2<br>Hap 2	This study
	Bursa	8	137- PV951931	Hap 4	This study
	Afyonkarahisar	11	143- PV951932	Hap 5	This study
	İstanbul	3	167- PV951939	Hap 8	This study
	Edirne	1	236- PV951942<br>237- PV951943<br>238- PV951944<br>239- PV951945<br>240- PV951946<br>241- PV951947<br>242- PV951948<br>243- PV951949	Hap 10<br>Hap 10<br>Hap 10<br>Hap 10<br>Hap 10<br>Hap 10<br>Hap 10<br>Hap 10	This study
	Antalya	12	263- PV951955<br>264- PV951956<br>265- PV951957<br>266- PV951958<br>267- PV951959	Hap 10<br>Hap 10<br>Hap 10<br>Hap 10<br>Hap 10	This study
	Tekirdağ	2	249- PV951950	Hap 11	This study
	Artvin	24	178- PV951940	Hap 9	This study
	Samsun	17	306- PV951976<br>307- PV951977<br>308- PV951978<br>309- PV951979	Hap 17<br>Hap 17<br>Hap 17<br>Hap 16	This study
	Greece		AJ504443	-	[72]
	Czechia		AY316340, AY316343	-	[33]
	Kazakhistan		AY426095	-	[33]
	Switzerland		AY663801	-	[73]
	Bulgaristan		AY582291	-	[33]
	İspanya		AY582292, AY582293	-	[33]
	Spain		DQ120851, DQ120853	-	[74]
	Fransa		AY663800	-	[73]
	Uşak, Türkiye		KF218396	-	[2]
	Samsun, Türkiye		KF218397	-	[2]
	Kırıkkale, Türkiye		KF218398	-	[2]
	Nevşehir, Türkiye		KF218399	-	[2]
	Konya, Türkiye		KF218402	-	[2]
	Slovakya		AY426098, KX537507	-	[33]
	Belgium		OQ939664, OQ939689, OQ939690	-	Horemans et al., unpubl
	Syria		AY316336, AY316337<br>AY426096	-	[11]
	Russia		AY426097	-	[33]
	Iran		EU084886, EU084887	-	[23]
	Iran		KF874512, KF874513	-	[75]
	Lebonan		EU084888, EU084889	-	[23]
	Cyprus		EU084891	-	[23]
	Jordan		JN087548-JN087550	-	[76]
	Jordan		KX375162	-	[77]

	Van, Türkiye		KF218400	-	[2]
	Trabzon,<br>Türkiye		KF218401	-	[2]
	Hatay, Türkiye		KF218403	-	[2]
	Tajikistan		PQ635183-PQ635186<br>PQ635190	-	[83]
	Azerbaijan		PP929216, PP929222<br>PP929206	-	[84]
	Armenia		PP929178, PP929132<br>PP929133, PP929135	-	[84]
	Malta		MN045612-MN045615	-	[78]
	France		EU420890-EU420892	-	Hulva ve Evin<br>unpubl
	Morocco		AY582283	-	[79]
	Morocco		AY582285	-	[33]
	Morocco		KM252778	-	[59]
	Sicily, Italy		EU084885	-	[23]
P. kuhlii	Adana	18	207- PV951866<br>208- PV951867<br>155- PV951868<br>175- PV951869<br>176- PV951870	Hap 1<br>Hap 2<br>Hap 3<br>Hap 3<br>Hap 3	This study
	Bitlis (Ahlat)	25	367- PV951871<br>368- PV951872<br>369- PV951873	Hap 2<br>Hap 4<br>Hap 4	This study
	Balıkesir	5	235- PV951874<br>363- PV951875<br>364- PV951876<br>365- PV951877<br>366- PV951878	Hap 2<br>Hap 2<br>Hap 2<br>Hap 2<br>Hap 2	This study
	Hatay	19	194- PV951879<br>195- PV951880<br>196- PV951881<br>197- PV951882<br>199- PV951883<br>201- PV951884	Hap 1<br>Hap 5<br>Hap 2<br>Hap 5<br>Hap 5<br>Hap 5	This study
	Kırşehir	14	181- PV951885<br>182- PV951886<br>183- PV951887<br>184- PV951888<br>185- PV951889	Hap 1<br>Hap 6<br>Hap 1<br>Hap 5<br>Hap 3	This study
	Mardin	22	222- PV951890<br>223- PV951891<br>224- PV951892<br>215- PV951893<br>217- PV951894<br>219- PV951895	Hap 2<br>Hap 2<br>Hap 2<br>Hap 2<br>Hap 2<br>Hap 2	This study
	Niğde	16	329- PV951896<br>330- PV951897<br>331- PV951898<br>332- PV951899<br>333- PV951900<br>334- PV951901<br>335- PV951902<br>336- PV951903	Hap 2<br>Hap 2<br>Hap 2<br>Hap 2<br>Hap 2<br>Hap 2<br>Hap 2<br>Hap 2	This study
	Sivas	20	304- PV951904	Hap 3	This study

	Şanlıurfa	21	173- PV951905<br>174- PV951906<br>209- PV951907	Hap 2<br>Hap 2<br>Hap 2	This study
	Şırnak	26	228- PV951908<br>229- PV951909<br>230- PV951910	Hap 2<br>Hap 2<br>Hap 4	This study
	Antalya, TR		KF218393	Hap 5	[2]
	Arabia		KC146388<br>KC146389<br>KC146390	Hap 7<br>Hap 2<br>Hap 8	[80]
	Azerbaijan		PP929193, PP929198, PP929199	Hap 2	[84]
	Cyprus		KP455370	Hap 1	
	Crete		KP455367, KP455368	Hap 9	[77]
	Egypt		KP455372	Hap 10	[77]
	Morocco		KM252759-KM252761, KM252765	Hap 12<br>Hap 9	[59]
	Southern Anatolia		KF218392	Hap 3	[2]
	Southeastern Anatolia		KF218390	Hap 2	[2]
	Hatay, TR		KF218395	Hap 2	[2]
	Iğdır, TR		KF218394	Hap 2	[2]
	Iran		KM252768-KM252772, KM252775	Hap 2<br>Hap 5	[59]
	Iran		AJ504445	Hap 5	[72]
	Jordan		KP455378-KP455381, KP455382, KP455384	Hap 2<br>Hap 5	[77]
	Kahramanmaras, TR		KF218391	Hap 2	[2]
	Libya		KM252756- KM252758	Hap 11	[59]
	Macedonia		AJ504444	Hap 9	[72]
	Syria		KM252766, KM252767	Hap 2	[59]
	Greece		KM252762, KM252763	Hap 9	[59]
P. nathusii	Muğla	7	99- PV951911	Hap 1	This study
	Çanakkale	4	132- PV951912	Hap 2	This study
	Bursa	8	134- PV951913<br>138- PV951914	Hap 2<br>Hap 3	This study
	Tekirdağ	2	157- PV951915<br>177- PV951918<br>246- PV951919<br>247- PV951920<br>250- PV951921	Hap 2<br>Hap 6<br>Hap 4<br>Hap 5<br>Hap 2	This study
	İstanbul	3	159- PV951916<br>160- PV951917	Hap 2<br>Hap 2	This study
	Belçika		OQ939775<br>OQ939781<br>OQ939788	Hap 2<br>Hap 7<br>Hap 2	[81]
	Spain		DQ120849<br>DQ120850	Hap 9<br>Hap 8	[74]
	Switzerland		AJ504446	Hap 2	[72]
P. pygmaeus	Balıkesir	5	358, 359	-	This study
	Çanakkale	4	107, 111, 146, 153	-	This study
	Edirne	1	236, 237, 238	-	This study
	Kastamonu	13	279, 280	-	This study

	Muğla	7	97, 98	-	This study
	Rize	23	291, 292, 293, 294	-	This study
	Sakarya	10	255	-	This study
	Samsun	17	306	-	This study
	Sinop	15	316, 317, 318	-	This study
	Azerbaijan		PP929194, PP929195, PP929203, PP929204	-	[84]
	Iran		EU084882, AY316321	-	[23]
	Greece		AY316330	-	[33]
	Greece		AJ504441	-	[72]
	Spain		AY582282	-	[33]
	Switzerland		AY663797	-	[73]
	Kırklareli, Türkiye		AY426087	-	[11]
	Cyprus		AJ504442	-	[72]
	Cyprus		EU084883, EU084884	-	[23]
	Spain		DQ120856, DQ120855	-	[74]
	Portugal		JX566938	-	[82]
	Czechia		AY316324, AY316323	-	[33]
**Outgroups**					
V. murinus			LC052295		
M. brandtii			AY665168		
M. albescens			PX273943		
M. myotis			AF376860		

Table S2. Genetic divergence (K2P), gene flow (Nm), and neutrality tests for *Pipistrellus* species based on Cyt*b* data. Nm < 1 indicates restricted gene flow; significant negative neutrality values denote expansion. ns = not significant.

Species / Lineage Comparison or Group	Mean Genetic Distance (K2P, %)	Gene Flow (Nm)	Tajima's D	Fu's Fs
Pipistrellus pipistrellus				
Group 1 vs Group 2	2.2 ± 0.40	0.16	—	—
Group 1 vs Mediterranean	4.5 ± 0.71	0.08	—	—
Group 2 vs Mediterranean	4.7 ± 0.72	0.12	—	—
Group 1	—	—	-1.170 (ns)	-16.766 (p < 0.02)
Group 2	—	—	-0.834 (ns)	4.646 (ns)
Pipistrellus kuhlii				
P. k. lepidus vs *P. k. kuhlii*	3.95 ± 0.76	0.35	—	—
P. k. kuhlii	—	—	1.254 (ns)	2.888 (ns)
P. k. lepidus	—	—	-1.850 (p < 0.05)	-2.042 (p < 0.05)
Pipistrellus nathusii				
Hap 1 vs Hap 2, 4-9	0.33 ± 0.16	—	—	—
Hap 1 vs Hap 3	0.29 ± 0.17	—	—	—
Hap 2, 4-9 vs Hap 3	0.22 ± 0.11	—	—	—
All samples (n = 11)	—	—	-2.152 (p < 0.05)	13.596

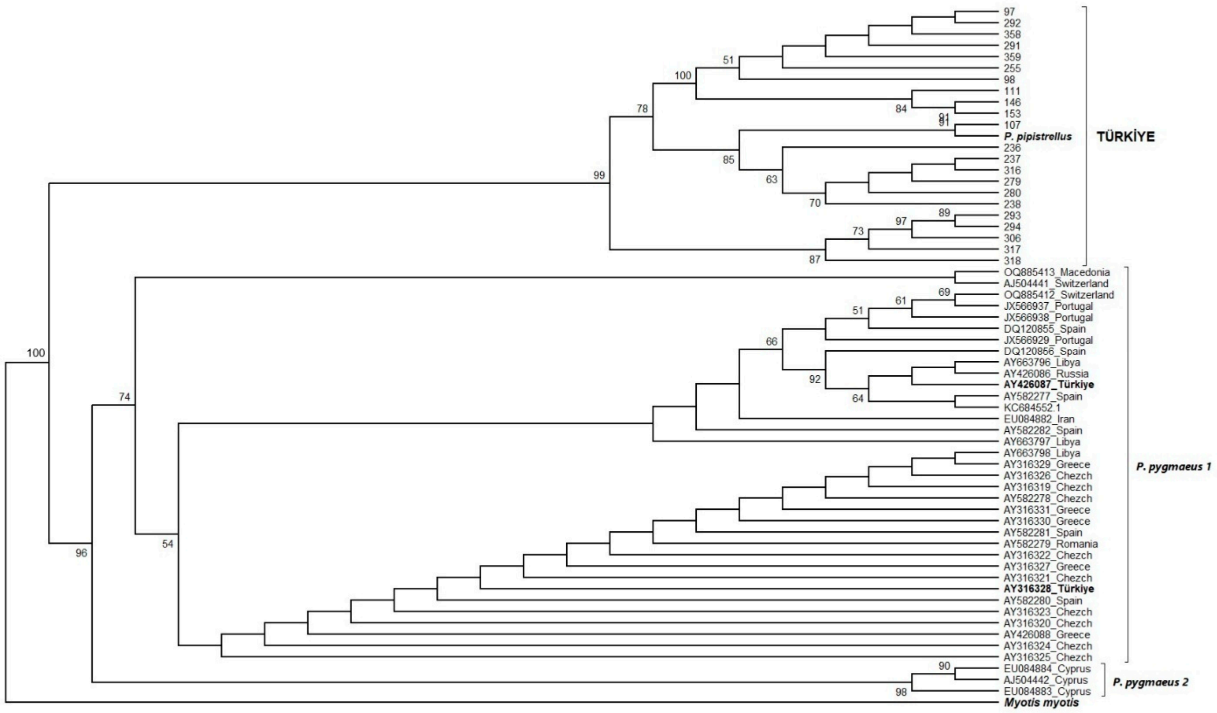

Figure S1. Maximum Likelihood (ML) phylogeny of Turkish specimens morphologically identified as *Pipistrellus pygmaeus*.
